# Knowledge, Attitude, and Practices Related to Foot Care Among Diabetic Patients in Tabuk City, Saudi Arabia

**DOI:** 10.7759/cureus.48473

**Published:** 2023-11-07

**Authors:** Tariq M Shaqran, Saud N Alqahtani, Abdullah F Alhalafi, Norah M Alsabeelah, Rafaa A Algethmi, Ammar S Azhari, Abdulrahman Y Alhashmi, Abdullah N Almaghrabi, Hibah A Alshammari, Mohammed Saeed Alshahrani

**Affiliations:** 1 Family Medicine, King Salman Armed Forces Hospital, Tabuk, SAU; 2 College of Medicine, King Abdulaziz University, Jeddah, SAU; 3 College of Medicine, University of Bisha, Bisha, SAU; 4 College of Medicine, University of Tabuk, Tabuk, SAU; 5 College of Medicine, Taibah University, Medinah, SAU; 6 Surgery, Prince Metaab Bin AbdelAziz Hospital, Aljouf, SAU

**Keywords:** type 1 diabetes, type 2 diabetes, diabetic foot, diabetes mellitus, amputation

## Abstract

Introduction

Diabetic foot ulcer (DFU) is a prevalent complication of diabetes mellitus (DM), affecting approximately 15% of all diabetic patients. This condition poses significant challenges due to its association with major morbidity, mortality, high costs, and diminished quality of life. The incidence of diabetic foot complications among diagnosed diabetes cases is alarming, making it a primary concern in diabetes management. Diabetes mellitus, a chronic metabolic disorder, impacts nearly every system in the body.

Methods

In this study, a cross-sectional design was employed to assess the level of knowledge, attitude, and practices related to foot care among 432 diabetic patients in Tabuk City, Saudi Arabia.

Results

The participants' ages ranged from 18 to above 60 years, with (n = 206, 47.69%) being male and (n = 226, 52.31%) female. Type 2 diabetes was prevalent, constituting (n = 277, 64.12%) of cases, whereas (n = 187, 38.29%) had type 1 diabetes. Approximately (n= 224, 51.9%) of patients had been diagnosed with diabetes for less than 10 years. A significant portion (n= 302, 69.91%) of patients did not report any foot complaints. However, (n= 88, 20.37%) had a history of healed ulcers, and (n= 21, 4.9%) had undergone amputation due to diabetes. The majority of patients (n = 228, 52.78%) were under oral agent treatment.

Conclusion

The study population demonstrated adequate knowledge about diabetes management and exhibited positive attitudes toward diabetes and its related complications, particularly concerning foot care. While most patients displayed appropriate practices related to diabetic foot care, some participants showed inadequate adherence to essential procedures. Addressing these gaps in knowledge and practices is crucial for enhancing the overall management of diabetic foot complications among patients.

## Introduction

Diabetes mellitus (DM) is a chronically debilitating medical condition that is spreading around the globe. According to the International Diabetes Federation Atlas, the prevalence of diabetes worldwide was predicted to be 9.3% (463 million people) in 2019, 10.2% (578.4 million) by 2030, and 10.9% (700.2 million) by 2045 [1,2]. Furthermore, according to the International Diabetes Federation Atlas, 18.3% of adult Saudis were estimated to have diabetes. It is associated with a higher incidence of disease, mortality, and growing healthcare costs. Diabetes dramatically raises the risk of several chronic conditions, such as heart disease, retinopathy, hypertension, and foot difficulties [3].

A crippling consequence of diabetes mellitus, diabetic foot disease eventually affects up to 50% of individuals with type 1 and type 2 diabetes. The affected patient's years of life and quality of life are still being significantly reduced as a result of this condition. Moreover, it accounts for at least 12-15% of the total expenses related to diabetes, and as much as 40% in underdeveloped nations. Furthermore, the diabetic foot disease treatments that are currently offered are typically not as successful as they should be [4,5]. This is mainly explained by the lack of understanding of its underlying mechanisms and available treatment options due to the low level of interest and funding for this complication's global research [6].

Diabetic foot significantly increases health care expenses, and improper foot care among diabetics is a primary cause of indisposition and early death. Overall, 3.3% of diabetic patients experienced foot problems; these included 2.05% foot ulcers, 0.19% gangrene, and 1.06% amputations [7]. On the other hand, some argue that teaching patients about the complications associated with diabetes and the importance of taking good care of their feet will lower the risk of complications, enhance the quality of life, and ultimately increase the financial burden on both the individual and society at large [8].

The Saudi Ministry of Health is facing a major dilemma because, according to its 2018 statistical yearbook, there were 1280 cases of amputation as a result of diabetes mellitus in males and 765 cases in females [3]. The foot issues are considered the most preventable of all diabetes-related consequences [9]. Proactive foot care and prevention are encouraged to lower the risk of amputation, costly resource utilization, and patient morbidity. Identifying risk factors, providing specialized podiatric care, and patient education are some of these strategies. It has been demonstrated that this strategy is both economical and efficient [10].

The incidence of diabetic foot ulcers and amputations can be effectively decreased by raising patient awareness, encouraging regular foot care routines, and helping diabetes patients maintain appropriate glycemic control [11]. Many scientific associations and organizations now offer guidelines for appropriate foot care education [12]. Thus, to improve awareness and encourage appropriate practices, the American Diabetic Association advised that all diabetic patients receive education regarding self-foot care [13].

The current study aimed to assess patients' knowledge and practices concerning diabetic foot care in Tabuk City, Saudi Arabia. As no similar study has been conducted in this area before, our research was designed to evaluate the level of knowledge and adherence to foot care practices among individuals diagnosed with diabetes mellitus. Implementing patient education strategies can significantly contribute to minimizing diabetic foot ulcers and amputations within the healthcare system.

## Materials and methods

Study design and area

In this cross-sectional study, our objective was to assess the knowledge, attitudes, and practices concerning foot care among diabetic patients in Tabuk City, Saudi Arabia. Data collection took place between July 10 and October 10, 2023, in diabetic clinics located within both civil and military hospitals. The selection of these clinics was done using a random sampling technique to ensure a representative sample for the study. Tabuk City is situated in the northwestern region of Saudi Arabia and is home to an estimated population of around 534,893 individuals, as per the latest data provided by the General Authority for Statistics in Saudi Arabia.

Study population and eligibility criteria

The study focused on diabetic patients aged 18 years and older, receiving care at diabetic centers in both civil and military hospitals in Tabuk City. Patients who were unable to provide the necessary information were excluded from the study. 

Sample size 

The sample size for this study was calculated using the following formula:

n = (Z^2 * P * Q) / d^2

Where: n = sample size; Z = z-score corresponding to the level of confidence desired (e.g., 1.96 for 95% confidence); P = expected prevalence of adequate knowledge and positive attitudes towards GDM management (assumed to be 50%); Q = 1 minus P; d = margin of error (assumed to be 5%). Assuming a 10% non-response rate, the final sample size was 432 diabetic patient

Sampling technique

Participants were chosen randomly utilizing a systematic random sampling method. In particular, every second individual receiving care at the diabetic centers during the study period was included. This method was employed to ensure the sample's representativeness, ensuring it closely mirrored the population, and to ensure a substantial number of participants for the study.

Data collection tools

A structured questionnaire with closed-ended questions was utilized for this study, adapted from a similar research conducted in Alkharj [14]. The questionnaire comprised four sections: demographic details, patients' understanding of diabetes, its complications, and management, patients' attitudes, and their practices (see Appendices). To maintain consistency in data collection, data collectors were trained in the Arabization of the questions through a dedicated workshop, thus minimizing potential variations in data collection methods.

Data analysis plan

For our analysis, we employed the Statistical Package for Social Sciences (SPSS) version 28 (IBM Corp., Armonk, NY). Descriptive statistics were used to compile and summarize the data. To explore the relationship between knowledge, attitudes, and practices concerning foot care among diabetic patients and demographic characteristics, we utilized the Chi-square test and logistic regression analysis. Statistical significance was determined by a p-value less than 0.05; any result below this threshold was regarded as statistically significant.

Ethical consideration

Ethical clearance for this study was obtained from the Institutional Review Board (IRB) of King Salman Armed Forces Hospital, as evidenced by approval number KSAFH-REC-2023-516. Before participating in the trial, all individuals gave oral informed consent. Stringent measures were implemented to guarantee the confidentiality and privacy of the participants.

## Results

Table 1 presents the demographic information of the participants, totaling 432 individuals, with (n = 226, 52.31%) females and (n = 206, 47.69%) males. The age range of participants varied from 18 years old to above 60 years old, and the majority were of Saudi nationality (n = 407, 94.21%).

**Table 1:** Sociodemographic characteristics of the study participants (n= 432) N: Number of participants; %: Percent of participants

Variables	Classifications	N	%
Gender	Female	226	52.31
	Male	206	47.69
Age groups (years)	18-30 years	128	29.63
	31-45 years	93	21.53
	46-60 years	155	35.88
	>60 years	56	12.96
Marital Status	Single	130	30.09
	Married	235	54.40
	Divorced	35	8.10
	Widowed	32	7.41
Education Level	Elementary school	19	4.40
	Middle school	28	6.48
	High school	131	30.32
	University	196	45.37
	Higher education	28	6.48
	No education	30	6.94
Occupation	Student	60	13.89
	Employed	154	35.65
	Unemployed	114	26.39
	Retired	104	24.07
Nationality	Saudi	407	94.21
	Non-Saudi	25	5.79
Smoking	Yes	113	26.16
	No	319	73.84

Table 2 outlines the clinical details of the participants. The majority of the study group had type 2 diabetes (n = 277, 64.12%). Patients with uncontrolled hemoglobin (Hb)A1c levels were higher (n = 187, 38.29%) compared to those with controlled HbA1c levels (n = 145, 33.56%), and a significant proportion of participants (n = 100, 23.15%) was unaware of their HbA1c readings. Among the participants, (n = 228, 52.87%) were on oral agents, while (n = 157, 36.43%) were on insulin treatment. A small percentage (n=27, 6.25%) relied solely on dietary management. Numbness emerged as the predominant foot problem (n = 140, 32.41%). Additionally, some participants reported other health issues, including amputation (n = 21, 4.86%), hypertension (n = 154, 35.65%), renal disease (n = 29, 6.71%), heart disease (n = 74, 17.13%), dyslipidemia (n = 191, 44.21%), and retinopathy (n = 119, 27.55%). Regarding diabetes management, (n = 295, 68.29%) of participants received advice on diabetes and its foot complications, while (n = 137, 31.71%) did not receive any guidance. Among those who received advice, physicians or healthcare professionals were the primary sources (n = 144, 33.33%).

**Table 2:** Clinical Information of the studied participants N: Number of participants; %: Percent of participants

Variables	Responses	N	%
Type of diabetes	Type 1	155	35.88
	Type 2	277	64.12
Duration of diabetes	≤10 years	224	51.85
	11-20 years	131	30.32
	21-30 years	37	8.56
	>30 years	40	9.26
The last reading of HbA1c	Controlled (<7.0%)	145	33.56
	Uncontrolled (7.0%-8.5%)	106	24.54
	Highly uncontrolled (≥8.6%)	81	18.75
	Unknown	100	23.15
What is your Diabetes treatment	Insulin	157	36.34
	Insulin and oral agent	20	4.63
	Oral agent (s)	228	52.78
	Diet	27	6.25
Foot complains	Current foot ulcer	51	11.81
	Flat foot	48	11.11
	None	302	69.91
	History of healed ulcer	88	20.37
Sensation problem in foot	Foot pain at rest (especially at night)	91	21.06
	Multiple problems	32	7.41
	Foot pain during walking	134	31.02
	Numbness	140	32.41
	None	176	40.74
Amputation	Yes	21	4.86
	No	411	95.14
Hypertension	Yes	154	35.65
	No	278	64.35
Renal disease	Yes	29	6.71
	No	403	93.29
Heart disease	Yes	74	17.13
	No	358	82.87
Dyslipidemia	Yes	191	44.21
	No	241	55.79
Retinopathy	Yes	119	27.55
	No	313	72.45
Received advice on foot care	Yes	295	68.29
	No	137	31.71
If yes, Source of advice	Internet / social media	35	8.10
	Multiple sources	55	12.73
	None	136	31.48
	Physicians / heath care	144	33.33
	Relative / friends	62	14.35

Table 3 demonstrates the participants' excellent knowledge of diabetes and its complications, as well as proper foot care practices for diabetic patients. In all the questions, the participants provided adequate answers exceeding 70%, indicating a high level of knowledge among the residents of Tabuk City.

**Table 3:** Knowledge responses of the participants N: Number of participants; %: Percent of participants

Variables	Responses	N	%
Diabetics are likely to develop foot ulcers	Yes	345	79.86
	No	87	20.14
Diabetics are likely to develop reduced blood flow in their feet	Yes	387	89.58
	No	45	10.42
Diabetics are likely to develop reduced sensation in their feet	Yes	394	91.20
	No	38	8.80
It is important to look at the soles because diabetics have reduced sensations	Yes	380	87.96
	No	52	12.04
It is important to inspect the feet, as wounds and infections may not heal quickly	Yes	386	89.35
	No	46	10.65
Poor circulation in feet may result from smoking	Yes	320	74.07
	No	112	25.93
It is important to look after the feet because they are more prone to be flat foot	Yes	326	75.46
	No	106	24.54
Taking medication regularly will reduce DM complication	Yes	380	87.96
	No	52	12.04
It is important to examine the inside of footwear for any object or tear	Yes	392	90.74
	No	40	9.26
Foot gangrene is one of the diabetic foot complications	Yes	392	90.74
	No	40	9.26
Do you think doing exercise will help you prevent diabetic foot?	Yes	340	78.70
	No	92	21.30
Uncontrolled diabetes can lead to foot deformity	Yes	331	76.62
	No	101	23.38

Table 4 presents the participants' attitudes toward managing diabetes. Responses regarding regular exercise and dietary changes to prevent further diabetic complications were favorable (n = 301, 69.68%). Additionally, participants displayed positive attitudes toward examining their feet and footwear, as well as practicing regular foot care (n = 282, 65.28%). Moreover, the majority of the participants exhibited a positive attitude (higher than 75%) toward the advice provided by specialist consultants concerning foot care in diabetes within the studied group.

**Table 4:** Attitudes responses of the participants N: Number of participants; %: Percent of participants

Variables	Responses	N	%
Can you perform regular exercise and change your food habits to prevent further diabetic complications?	Yes	301	69.68
	No	131	30.32
Can you take the responsibility of daily examination of your feet and footwear, as well as regular foot-care specialist consultation?	Yes	282	65.28
	No	150	34.72
Can you use special footwear advised by the foot-care specialist?	Yes	362	83.80
	No	70	16.20
Will you wear footwear indoors as advised by your foot-care specialist?	Yes	334	77.31
	No	98	22.69
Can you be able to live a normal life with appropriate measures for diabetes?	Yes	362	83.80
	No	70	16.20

Table 5 outlines the participants' practices related to diabetes management. It was observed that a significant number of participants, (n= 227, 52.55%), did not examine their feet daily, indicating an inadequate response in this aspect. Similarly, a considerable portion of the participants (n = 260, 60.19%) did not regularly visit a physician for foot check-ups, indicating a lack of regular medical monitoring. However, participants demonstrated positive practices in response to other questions, with more than 60% showing a favorable approach.

**Table 5:** Practices responses of the participants N: Number of participants; %: Percent of participants

Variables	Responses	N	%
Do you examine your feet daily?	Yes	205	47.45
	No	227	52.55
Do you use comfortable, closed, and soft footwear?	Yes	319	73.84
	No	113	26.16
Do you examine your shoes before wearing them?	Yes	262	60.65
	No	170	39.35
Do you walk barefoot, outside?	Yes	102	23.61
	No	330	76.39
Do you continuously wear cotton socks?	Yes	208	48.15
	No	224	51.85
Do you change your socks daily?	Yes	300	69.44
	No	132	30.56
Do you examine your feet for any marks resulting from shoes/socks?	Yes	304	70.37
	No	128	29.63
Do you daily wash your feet with warm water?	Yes	331	76.62
	No	101	23.38
Do you carefully dry the cleft between toes after washing?	Yes	249	57.64
	No	183	42.36
Do you apply moisturizer daily on your feet?	Yes	226	52.31
	No	206	47.69
Do you cut your toenails regularly?	Yes	344	79.63
	No	88	20.37
Do you regularly visit a physician for foot check-ups?	Yes	172	39.81
	No	260	60.19
Do you regularly change footwear, even without damage?	Yes	267	61.81
	No	165	38.19

In analyzing the association between participants' knowledge, attitudes, and practices as presented in Table 6, certain observations were made. Notably, no significant correlations were found for statements such as awareness about decreased sensation in diabetic feet, smoking's impact on foot circulation, and the need to inspect shoes for objects or tears. Similarly, foot gangrene awareness, assuming responsibility for regular podiatric visits, wearing specifically recommended shoes, opting for soft, closed, and comfortable footwear, going barefoot outdoors, and using warm water for foot washing showed no significant relationships. Additionally, regular toenail cutting and doctor visits, along with replacing shoes despite no damage, displayed no significant correlation. However, highly significant correlations were observed for recognizing decreased foot blood flow due to diabetes (P < 0.001), emphasizing the importance of foot care for flat feet (p = 0.07), leading a normal life with proper diabetes management (p = 0.001), daily foot checks, inspecting shoes daily (p < 0.001), frequent sock replacement (p = 0.08), inspecting feet for scuffs, and moisturizing feet daily (p < 0.001). These results highlight critical areas where diabetic patients' awareness and practices intersect, shedding light on essential aspects of foot care.

**Table 6:** Association between knowledge, attitude, and practices of the participants and their responses toward the study variables

Variables	Responses Of the participants	Female No. 226 (52.3%)	Male No. 206 (47.7%)	p value Fisher’s exact test
Knowledge				
Diabetics are likely to develop foot ulcers	Yes	173 (76.5%)	172 (83.5%)	0.046
	No	53 (23.5%)	34 (16.5%)	
Diabetics are likely to develop reduced blood flow in their feet	Yes	214 (94.7%)	173 (84%)	<0.001
	No	12 (5.3%)	33 (16%)	
Diabetics are likely to develop reduced sensation in their feet	Yes	211 (93.4%)	183 (88.8%)	0.068
	No	15 (6.6%)	23 (11.2%)	
It is important to look at the soles because diabetics have reduced sensations	Yes	205 (90.7%)	175 (85%)	0.046
	No	21 (9.3%)	31 (15%)	
It is important to inspect the feet, as wounds and infections may not heal quickly	Yes	209 (92.5%)	177 (85.9%)	0.020
	No	17 (7.5%)	29 (14.1%)	
Poor circulation in feet may result from smoking	Yes	170 (75.2%)	150 (72.8%)	0.323
	No	56 (24.8%)	56 (27.2%)	
It is important to look after the feet because they are more prone to be flat foot	Yes	182 (80.5%)	144 (69.9%)	0.007
	No	44 (19.5%)	62 (30.1%)	
Taking medication regularly will reduce DM complication	Yes	205 (90.7%)	175 (85%)	0.046
	No	21 (9.3%)	31 (15%)	
It is important to examine the inside of footwear for any object or tear	Yes	204 (90.3%)	188 (91.3%)	0.425
	No	22 (9.7%)	18 (8.7%)	
Foot gangrene is one of the diabetic foot complications	Yes	210 (92.9%)	182 (88.3%)	0.071
	No	16 (7.1%)	24 (11.7%)	
Do you think doing exercise will help you prevent diabetic foot?	Yes	186 (82.3%)	154 (74.8%)	0.036
	No	40 (17.7%)	52 (25.2%)	
Uncontrolled diabetes can lead to foot deformity?	Yes	178 (78.8%)	153 (74.3%)	0.162
	No	48 (21.2%)	53 (25.7%)	
Attitude				
Can you perform regular exercise and change your food habits to prevent further diabetic complications?	Yes	168 (74.3%)	133 (64.6%)	0.018
	No	58 (25.7%)	73 (35.4%)	
Can you take the responsibility of daily examination of your feet and foot-wear, as well as regular foot-care specialist consultation?	Yes	154 (68.1%)	128 (62.1%)	0.113
	No	72 (31.9%)	78 (37.9%)	
Can you use special foot-wear advised by the foot-care specialist?	Yes	195 (86.3%)	167 (81.1%)	0.090
	No	31 (13.7%)	39 (18.9%)	
Will you wear footwear indoors as advised by your foot-care specialist?	Yes	183 (81%)	151 (73.3%) %	0.037
	No	43 (19%)	55 (26.7%)	
Can you be able to live a normal life with appropriate measures for diabetes?	Yes	202 (89.4%)	160 (77.7%)	<0.001
	No	24 (10.6%)	46 (22.3%)	
Practices				
Do you examine your feet daily?	Yes	128 (56.6%)	77 (37.4%)	<0.001
	No	98 (43.4%)	129 (62.6%)	
Do you use comfortable, closed, and soft footwear?	Yes	173 ((76.5%)	146 (70.9%)	0.109
	No	53 (23.5%)	60 (29.1%)	
Do you examine your shoes before wearing them?	Yes	157 (69.5%)	105 (51%)	<0.001
	No	69 (30.5%)	101 (49%)	
Do you walk barefoot, outside?	Yes	48 (21.2%)	54 (26.2%)	0.135
	No	178 (78.8%)	152 (73.8%)	
Do you continuously wear cotton socks?	Yes	109 (48.2%)	99 (48.1%)	0.524
	No	117 (51.8%)	107 (51.9%)	
Do you change your socks daily?	Yes	169 (74.8%)	131 (63.6%)	0.008
	No	57 (25.2%)	75 (36.4%)	
Do you examine your feet for any marks resulting from shoes/socks?	Yes	178 (78.8%)	126 (61.2%)	<0.001
	No	48 (21.2%)	80 (38.8%)	
Do you daily wash your feet with warm water?	Yes	175 (77.4%)	156 (75.7%)	0.380
	No	51 (22.6%)	50 (24.3%)	
Do you carefully dry the cleft between toes after washing?	Yes	138 (61.1%)	111 (53.9%)	0.079
	No	88 (38.9%)	95 (46.1%)	
Do you apply moisturizer daily on your feet?	Yes	147 (65%)	79 (38.3%)	<0.001
	No	79 (35%)	127 (61.7%)	
Do you cut your toenails regularly?	Yes	187 (82.7%)	157 (76.2%)	0.059
	No	39 (17.3%)	49 (23.8%)	
Do you regularly visit a physician for foot check-ups?	Yes	98 (43.4%)	74 (35.9%)	0.069
	No	128 (56.6%)	132 (64.1%)	
Do you regularly change footwear, even without damage?	Yes	148 (65.5%)	119 (57.8%)	0.061
	No	78 (34.5%)	87 (42.2%)	

## Discussion

Diabetic foot disease stands out as a prevalent and potentially fatal complication of diabetes mellitus (DM). Its persistence often leads to significant morbidity and premature mortality. The American Diabetes Association underscores the importance of an annual comprehensive foot examination for individuals with diabetes. Diligent foot care can effectively prevent the majority of foot-related complications. Although cultivating meticulous foot care practices demands dedication and time, self-care remains pivotal in averting potential problems.

This study enrolled 432 diabetic patients, with 64.2% (n = 277) diagnosed with type 2 diabetes, and the remaining 35.88% (n = 155) diagnosed with type 1 diabetes. The age range of the participants spanned from 18 to above 60 years. Among them, 52.31% (n = 226) were female, and 47.69% (n = 206) were male. The majority of the participants were of Saudi nationality, accounting for 94.21% (n = 407), while 5.79% (n = 25) were non-Saudi patients.

The majority of patients did not report foot complaints (n = 302, 69.91%). A total of 21 patients (4.6%) had undergone amputation, while 29 patients (6.71%) had renal diseases. Heart diseases were present in 74 patients (17.13%), dyslipidemia was observed in 191 patients (44.21%), and retinopathy was found in 119 patients (27.55%). Among the participants, 224 individuals (51.85%) had diabetes for 10 years or less. Uncontrolled HbA1c levels were predominant, with 187 patients (43.29%), and the majority of participants were undergoing oral agent treatment (n = 228, 52.87%).

This study demonstrated that diabetic patients possessed a high level of knowledge concerning diabetes, its complications, and appropriate foot care practices, with over 70% of participants providing accurate responses to all questions. These findings contrast with studies conducted by Taksande et al., where patients displayed poor knowledge about diabetes and its complications [15], and another study in Iran in 2020, where only 15.2% of patients exhibited adequate knowledge about diabetes [16]. However, our results align with a study conducted in Riyadh, Saudi Arabia, by Alshammari et al., which reported a good knowledge rate of 76.6% among the participants [17].

Our study found that the primary source of information for the participants was physicians and healthcare providers (n = 144, 33.3%). This percentage was notably higher than the 22% reported by Alshammari et al. [17] and significantly surpassed the 16.6% reported in other studies [18].

Participants exhibited more positive attitudes toward managing their diabetes than anticipated. Responses indicating regular exercise and dietary modifications to prevent further complications from diabetes were satisfactory (n = 301, 69.68%), as were those related to regular foot care and the examination of feet and footwear (n = 282, 65.28%). Moreover, participants displayed positive attitudes, with more than 75% endorsing the advice of specialist consultants regarding foot care in diabetes. These results align with a similar study conducted in the Aseer Region of Saudi Arabia [19].

Our study revealed inadequate practices among the participants, notably in the daily examination of their feet, with 227 participants (52.55%) failing to inspect their feet daily. Additionally, participants exhibited insufficient adherence to regular physician foot check-ups, as 260 patients (60.19%) did not visit physicians for regular foot examinations. Similar findings were reported by Vighnesh et al. [20] and supported by Al Amri et al. [19] in their respective studies.

Regarding the association between the knowledge, attitude, and practices of the participants and their responses, we observed varied associations, with approximately 47% exhibiting an association and nearly 53% lacking association. Female participants demonstrated higher knowledge levels than males on several aspects: understanding that diabetics can experience reduced blood flow (the female percentage was 94.75%, compared to 84% in males, p=<0.001), recognizing that diabetics may have reduced sensations (female association was 90.7%, compared to 85% in males, p=0.046), understanding the importance of inspecting the feet (92.5% in females compared to 85.9% in males, p=0.02), recognizing the significance of foot care due to the increased likelihood of flat feet (female association was 80.5% compared to 69.9% in males, p=0.007), acknowledging that regular medication intake reduces diabetes complications (90.7% in females compared to 85% in males, p=0.046), and understanding that exercise aids in preventing diabetic foot problems (82.3% in females compared to 74.8% in males, p=0.036). No significant gender differences were noted in other knowledge variables, consistent with findings from previous studies [20-22].

Our findings align with prior research, particularly concerning attitudes where women demonstrated higher associations than men. In terms of engaging in regular exercise to prevent further diabetes complications, women exhibited associations of 74.3%, compared to 64.6% in men (p = 0.018). Similarly, concerning wearing footwear indoors as recommended by foot care specialists, women's associations were 81%, while men's associations were 73.3% (p = 0.037). Additionally, regarding the ability to lead a normal life with appropriate diabetes measures, women's responses were 89.4%, surpassing men's responses at 77.7% (p = 0.001) [16,23]. These results emphasize the gender disparities in attitudes toward diabetic foot care, highlighting the need for targeted educational interventions tailored to both genders.

In terms of practices, similar patterns were observed, with female associations significantly higher than males. Regarding daily foot examination, 56.6% of females responded affirmatively compared to 37.4% of males (p=0.001). When it came to checking shoes before wearing them, 69.5% of females practiced this habit, while only 51% of males did so (p=0.001). Changing socks daily was more prevalent among females, with 74.8% following this practice compared to 63.6% of males (p=0.008). Inspecting feet for marks resulting from shoes or socks was more common among females, with a response rate of 78.8%, while only 61.2% of males reported this practice (p=0.001). Additionally, the application of daily moisturizer on feet exhibited a significant gender difference, with 65% of females adhering to this practice, contrasting with 38.3% of males (p=0.001). These findings corroborate with studies by Navarro-Peternella et al. [24] and Ciarambino et al. [25], highlighting consistent gender disparities in diabetic foot care practices and emphasizing the need for targeted interventions to bridge these gaps.

Limitations of the study

The study's questionnaire design had limitations as it only allowed for yes or no responses to questions about knowledge, attitude, and practices, which can be considered a drawback. While closed-ended questions simplify participant responses, they might have led to affirmative answers when participants were unsure, potentially inflating the perceived knowledge and attitude levels. To mitigate this bias, incorporating a "how often they should do it" would give more precise insight into their attitude and practices. Additionally, the study lacked information about participants' family history of diabetes, a factor important in understanding the genetic predisposition to type 2 diabetes. Genetic factors, including various gene mutations, have been associated with diabetes development. Addressing these limitations in future research can enhance the comprehensiveness of the study and provide a more nuanced understanding of the factors influencing participants' knowledge, attitudes, and practices related to diabetes management.

## Conclusions

The study's findings highlight the need for targeted foot care interventions for patients with diabetes mellitus. In summary, our research revealed that nearly two-thirds of the diabetic patients studied possessed a strong understanding of diabetic foot issues. Furthermore, patients exhibited positive attitudes regarding the management of diabetic feet and the implications of diabetes on foot health. However, both the practice of daily foot checks and participants' willingness to consult a doctor for regular check-ups were insufficiently addressed. Despite these gaps, the Tabuk community demonstrates a commendable level of awareness, attitude, and practice concerning diabetes.

## Appendices

**Table 7:** Study questionnaire

Section 1: Demographic Information	
Variables	Classifications
Gender	Female
	Male
Age groups (years)	18-30 years
	31-45 years
	46-60 years
	>60 years
Marital Status	Single
	Married
	Divorced
	Widowed
Education Level	Elementary school
	Middle school
	High school
	University
	Higher education
	No education
Occupation	Student
	Employed
	Unemployed
	Retired
Nationality	Saudi
	Non-Saudi
Smoking	Yes
	No
Section 2: Knowledge	
Diabetics are likely to develop foot ulcers	Yes
	No
Diabetics are likely to develop reduced blood flow in their feet	Yes
	No
Diabetics are likely to develop reduced sensation in their feet	Yes
	No
It is important to look at the soles because diabetics have reduced sensations	Yes
	No
It is important to inspect the feet, as wounds and infections may not heal quickly	Yes
	No
Poor circulation in feet may result from smoking	Yes
	No
It is important to look after the feet because they are more prone to be flat foot	Yes
	No
Taking medication regularly will reduce DM complication	Yes
	No
It is important to examine the inside of footwear for any object or tear	Yes
	No
Foot gangrene is one of the diabetic foot complications	Yes
	No
Do you think doing exercise will help you prevent diabetic foot?	Yes
	No
Uncontrolled diabetes can lead to foot deformity?	Yes
	No
Section 3: Attitude	
Can you perform regular exercise and change your food habits to prevent further diabetic complications?	Yes
	No
Can you take the responsibility of daily examination of your feet and foot-wear, as well as regular foot-care specialist consultation?	Yes
	No
Can you use special foot-wear advised by the foot-care specialist?	Yes
	No
Will you wear footwear indoors as advised by your foot-care specialist?	Yes
	No
Can you be able to live a normal life with appropriate measures for diabetes?	Yes
	No
Section 4: Practices	
Do you examine your feet daily?	Yes
	No
Do you use comfortable, closed, and soft footwear?	Yes
	No
Do you examine your shoes before wearing them?	Yes
	No
Do you walk barefoot, outside?	Yes
	No
Do you continuously wear cotton socks?	Yes
	No
Do you change your socks daily?	Yes
	No
Do you examine your feet for any marks resulting from shoes/socks?	Yes
	No
Do you daily wash your feet with warm water?	Yes
	No
Do you carefully dry the cleft between toes after washing?	Yes
	No
Do you apply moisturizer daily on your feet?	Yes
	No
Do you cut your toenails regularly?	Yes
	No
Do you regularly visit a physician for foot check-ups?	Yes
	No
Do you regularly change footwear, even without damage?	Yes
	No
